# Tissue Engineering in Pediatric Bladder Reconstruction—The Road to Success

**DOI:** 10.3389/fped.2019.00091

**Published:** 2019-03-29

**Authors:** Maya Horst, Daniel Eberli, Rita Gobet, Souzan Salemi

**Affiliations:** ^1^Laboratory for Urologic Tissue Engineering and Stem Cell Therapy, Department of Urology, University Hospital, Zurich, Switzerland; ^2^Division of Pediatric Urology, Department of Pediatric Surgery, University Children‘s Hospital, Zurich, Switzerland

**Keywords:** myelomeningocele, neurogenic bladder, bladder augmentation, tissue engineering, stem cells, pediatric

## Abstract

Several congenital disorders can cause end stage bladder disease and possibly renal damage in children. The current gold standard therapy is enterocystoplasty, a bladder augmentation using an intestinal segment. However, the use of bowel tissue is associated with numerous complications such as metabolic disturbance, stone formation, urine leakage, chronic infections, and malignancy. Urinary diversions using engineered bladder tissue would obviate the need for bowel for bladder reconstruction. Despite impressive progress in the field of bladder tissue engineering over the past decades, the successful transfer of the approach into clinical routine still represents a major challenge. In this review, we discuss major achievements and challenges in bladder tissue regeneration with a focus on different strategies to overcome the obstacles and to meet the need for living functional tissue replacements with a good growth potential and a long life span matching the pediatric population.

## Introduction

Congenital disorders such as posterior urethral valves, bladder extrophy, and neurogenic bladder result in reduced bladder capacity, impaired compliance, incontinence, and possibly renal damage. Despite decades of experience in the management of end stage bladder disease, current therapy options are not curative. Enterocystoplasty—bladder augmentation using an intestinal segment—is the gold standard therapy if medical management fails. However, it is associated with severe complications, including metabolic disturbances, stone formation, urine leakage, and chronic infections owing to the inherent absorptive and secretory properties of the gastrointestinal segments (1–3). Given the limited success and high morbidity with current treatment options, tissue engineering (TE) has been considered as a novel treatment approach. The regeneration of bladder tissue derived from the patient's own cells may represent an attractive option particularly for patients of pediatric urology. The pediatric population presents several opportunities for the application of TE, as the regenerative capacity is significantly greater in infants and children than in adults. However, the specific needs of the pediatric population, primarily the need for living functional tissue replacements with a good growth potential and a long life span need to be addressed.

First attempts to replace bladder tissue by synthetic materials were performed in the 1950s, where plastic urinary bladder substitutes were implanted into patients (4, 5). The first biomaterials used for reconstruction of the urinary bladder for clinical applications were gelatin sponge (6, 7), cellular collagen biomatrix (8) resin sprayed paper (9), bovine pericardium (10), and dura (11). However, due to unsatisfactory postoperative results this technique was suspended.

The urinary bladder possesses a unique anatomy, allowing for repetitive expansion, and contraction and withstanding the urine pressure. Furthermore, the bladder is lined with a highly specialized multilayer epithelium, the urothelium, which acts as a tight urine barrier (12). The complexity of this structure poses a challenge for regenerative medicine. During the last two decades, TE has become a rapidly growing field of research in biotechnology and medicine. It is driven by the fascinating idea of generating autologous tissue substitutes for the treatment of tissue defects and organ failure. Several animal studies have shown promising results in bladder TE. The concept of TE involves the integration of various interacting components: the applied cells need to be held together by a tridimensional scaffold which provides the shape and initial mechanical strength, and molecular signals need to induce tissue regeneration *in vivo*. There are two common approaches in bladder engineering. The acellular approach includes the use of natural or synthetic biomaterials to enhance the body's natural growth to regenerate and repair itself (13). In the cellular approach, the removed donor tissue is dissociated into individual cells, either mechanically and by enzymatic digestion. Subsequently, the functional cells are either directly implanted into the host or seeded on a suitable biomaterial after expansion in culture and thereafter implanted into a non-functional site of the bladder (14).

Several cell based approaches (15–17) in different animal models (18–20) were successful with the formation of native tissue-like epithelialization and progressive muscle and blood vessel formation (21). Atala's group created engineered bladder tissue in a canine model using autologous cells and showed functional and anatomical characteristics of a normal bladder (3, 22). In a first clinical study human bladder was engineered for patients (aged 4–19 years) with end-stage bladder disease by isolating patient's autologous bladder urothelial and muscle cells, expanding the cells *in vitro*, and seeding them on a biodegradable collagen-polyglycolic acid scaffolds. The implanted composite engineered bladders were reported to show sustainably improved functional parameters (23). However, the majority of treated patients did not achieve good bladder capacity and compliance, but developed fibrosis of the artificial bladder wall. A recent phase II study (24), using an autologous cell seeded biodegradable composite scaffold for augmentation cystoplasty in children with spina bifida did not provide improved bladder compliance or capacity. Even though these two clinical trials were similar in design and used the same cell types, the differences in cell number, type of biomaterial, or surface area grafted and type of surgery might have influenced the outcome. To date, clinical translation has failed to establish a reliably effective treatment. Various hurdles such as early tissue fibrosis, lack of vascularization, insufficient urine barrier and inadequate contractibility are challenges encountered in regenerative medicine (25, 26). In this review, we discuss the major achievements and challenges in bladder tissue regeneration and focus on different strategies to overcome obstacles.

## Cellular Application in Tissue Engineering

Autologous cells are a perfect match for bladder engineering as they don't provoke inflammation and immune rejection, which are adverse effects of non-self donor cells. They can be derived from bladder tissue or from stem cells of another origin, such as the bone marrow or adipose tissue, however research in this regard is still less advanced (Table 1). Urinary bladder is a hollow organ composed of smooth muscle, urothelium, lamina propia, extracellular matrix, nerves, and vessels. Since detrusor muscle and urothelium represent the main properties of the bladder, the main focus of attention for bladder engineering was directed toward cells originating from these two tissues. Normal human bladder urothelium and muscle cells have been isolated from biopsies, expanded in culture, and characterized regarding differentiation characteristics and other biological functions (15, 42–44). The successful use of autologous cells for human bladder engineering derived from patients with end-stage bladder disease was shown by Atala et al. (23) and Joseph et al. (24). However, the use of autologous cells presupposes the availability of viable cells devoid of any genetic defect within the damaged or diseased tissue. In case of infection, altered tissue composition or malignancy, the adult autologous cells may be abnormal. Moreover, biopsies can lead to several problems including donor site morbidity, limited sample size, restricted proliferation ability, and loss of contractile phenotype of the cells during *in vitro* culture and expansion (45).

**Table 1:** Bladder tissue regeneration with cell therapy in human and animals.

**Study**	**Cell types**	**Biomaterial**	***In vivo* model**	**Remarks**
Atala et al. (14)	UC	PGA	Mouse Implantation into mesentery, omentum, retroperitoneum	Successful harvesting, culturing, and seeding of UC.
Cilento et al. (15)	Human bladder UC	PGA	Mouse Subcutaneous implant	Formation of multilayered structures
Yoo et al. (22)	SMC, UC	BAM	Beagle dogs Cystoplasty	Normal bladder compliance and increase in capacity compared with unseeded controls
Oberpenning et al. (3)	Autologous SMC, UC	PLGA coated PGA	Beagle dogs Cystoplasty	Normal capacity, elastic properties, and histologic architecture of the bladder wall.
Schoeller et al. (18)	UC	Silicon	Rat Cystoplasty	Successful use of vascularized prefabricated flaps for cystoplasty in animals with better survival rate compared to control groups.
Lai et al. (17)	Human SMCs from normal, exstrophic. neurogenic bladders	Unwoven PGA	Mouse Subcutaneous implant	Engineered muscle from normal and diseased bladders retain their phenotype *in vitro* and *in vivo* with the same degree of contractility regardless of their origin.
Fraser et al. (19)	Pig UC	Polyglactin carrier meshes and deepithelialized autologous colon	Minipigs Cystoplasty	Significant contraction and poor urothelial coverage.
Lakshmanan et al. (27)	hEG, SMC, UC	SIS	None	Co-cultured hEG cells grew well *in vitro*.
Frimberger et al. (28)	Human ESC SMCs, UC	SIS	Rat Cystoplasty	Improved regeneration of the ESC-seeded grafts compared to unseeded SIS.
Chung et al. (29)	BMSC	SIS	Rat Cystoplasty	More rapid tissue reconstitution compared to unseeded controls.
Jack et al. (30)	Human PLA cells	-	Rat, mouse Injection into urethral and bladder wall	Smooth muscle regeneration and phenotypic differentiation
Zhang et al. (31)	BMSC	SIS	Dog Cystoplasty	BMSC-seeded SIS scaffold promoted bladder regeneration.
Atala et al. (23)	Autologous UC, SMC	PGA	Human Cystoplasty	The engineered bladders showed improved functional parameters over a short period (10 months).
Jack et al. (32)	Human ADSC	PLGA	Rat Cystoplasty	Short-term improvement in physical properties of engineered bladder tissue.
Sakuma et al. (33)	Adipocytes	-	Mouse Injection to the cryo-injured bladder wall	Adipocytes differentiated into SMC lineages and contributed to the bladder wall regeneration.
Bodin et al. (34)	USCs	bacterial cellulose polymer	Mouse Subcutaneous implant	Differentiated USCs expressed urothelial and SMC markers.
Adamowicz et al. (35)	BMSC	Human amniotic membrane /tachosil sponge	Rat Cystoplasty	Formation of an autonomic SMC population poorly integrated into the bladder wall.
Horst et al. (36)	SMC	PLGA/BAM Hybrid scaffold	Rat Cystoplasty	Bladder regeneration with improved bladder architecture (urothelium, smooth muscle and collagen rich layers, micro vessels) in hybrid compared to BAM only scaffolds.
Imbeault et al. (37)	DF, HUVEC, UC	DF sheets	Mouse Subcutaneous implantation	Good vascularization, with capillary-like structures in the whole thickness of the tubes.
Joseph et al. (24)	Autologous SMC, UC	Polyglycolide/polylactide mesh	Human Cystoplasty	Cell seeded scaffold did not improve bladder compliance or capacity.
Horst et al. (38)	SMC	PLGA/BAM Hybrid scaffold	Rat Cystoplasty	Increased porosity enhanced cell proliferation *in vitro* and tissue ingrowth *in vivo*.
Lee et al. (39)	USC	heparin-immobilized bFGF-loaded scaffold	Rat Cystoplasty	Cell seeded scaffolds significantly increased bladder capacity, compliance, regeneration of smooth muscle tissue, multi-layered urothelium.
Zhe et al. (40)	ADSC	BAM	Rat Cystoplasty	Morphological regeneration of the bladder smooth muscle and nerves. Improvement of bladder capacity.
Horst et al. (41)	SMC	Polyesterurethane/BAM Hybrid scaffold	Rat Cystoplasty	Bladder tissue formation with excellent tissue integration and low inflammatory reaction.

*ADSC, Adipose derived stem cells; BAM, pig bladder derived acellular; BMSC, bone marrow stem cells; DF, Dermal fibroblasts; ESC, Embryonic stem cells; EnSCs, Endometrial stem cells; hEG, Human embryonic germ cells-derived stem cells; HUVEC, Human umbilical vein endothelial cells; PGA, polyglycolic acid polymer; PLA, Processed lipoaspirate; PLGA, poly lactic-co-glycolic acid; SIS, porcine small intestinal submucosa; SMC, smooth muscle cells; UC, Urothelial cells; USC, Urine-derived stem cells*.

Smooth muscle cells (SMCs) play an important role in the functionality of the bladder and both good proliferation potential and contractile function are essential for successful tissue regeneration. Unfortunately, even mature SMCs isolated from healthy sources have shown limited proliferation capacity and loss of the contractile phenotype followed by a change to a synthetic form during *in vitro* expansion (45). The phenotypic switch between a synthetic (proliferative) or contractile (quiescent) but active phenotype can occur reversibly and transiently *in vitro* and *in vivo* (46, 47). SMCs derived from neuropathic bladders have been shown to retain their pathological characteristics *in vitro* (48). Therefore, to overcome these limitations, embryonic (27), adult, and induced pluripotent stem cells (49) have been considered for bladder engineering.

In order to create clinically applicable engineered bladder tissue using stem cells, distinct selection criteria such as accessibility with minimal invasiveness, the ability to yield large number of cells in a limited time frame, only minor changes during *in vitro* culturing, reproducibility with a high differentiation potential are mandatory. Therefore, the type and quality of stem cells for bladder engineering are critical factors.

Embryonic stem cells (ESCs) can be isolated from the blastocyst inner cell mass. They are pluripotent cells with the ability to differentiate into any cell type and with an unlimited expansion potential *in vitro* (50, 51). Recently, ESC were differentiated to mesenchymal like stem cells (MSCs) by differentiation with growth factor cocktails and supporting feeder cells (OP9) (52). ESC can be induced to become SMCs under retinoic acid treatment, expressing SMC gene markers (53–55). Therefore, they are a valuable tool to study the differentiated SMC and to test their response to therapeutic agents. In a recent study using a rat model, MSCs derived from human ESCs were shown to more effectively improve the contractile function and the potential to repair the histological injury in interstitial cystitis/bladder pain syndrome than adult bone-marrow derived cells (56). The co-culture of human ESCs with bladder SMCs and urothelium seeded on porcine small intestinal submucosa (SIS) generated viable grafts *in vitro* (27). In a follow up study, the same construct was used to augment a previously injured rat bladder, resulting in an improved regeneration of the ESC-seeded graft compared to unseeded SIS (28). However, several safety issues such as the formation of teratoma, potential immune reactions, and the risk of differentiating into unwanted cell types limit their applicability for bladder engineering.

The ability of adult stem cells to differentiate and self-renew makes them a suitable source for bladder engineering. The adult stem cells can be isolated from virtually every tissue and organ type in mammals (57). Several adult stem cell types with different availabilities are currently used for bladder bioengineering, including adipose derived stem cells (ADSCs) (58), bone marrow stem cells (29), endometrial cells, menstrual blood cells and urine derived stem cells (UDSCs).

Human ADSCs have several advantages in TE applications due to their mutipotency, ease of access and high proliferative potential. They can be isolated either from subcutaneous fat tissue biopsies or by liposuction; both procedures are less invasive and painful than bone marrow aspiration. Human ADSC have surface antigens similar to MSCs derived from human bone marrow stromal cells (58). Several studies have shown efficient differentiation of ADSCs to SMCs and urothelial cells when placed in specific induction media (59–61). In a rat model, Jack et al. (30) delivered human processed lipoaspirate cells into the bladder and urethra. The cells remained viable for up to 12 weeks, showed evidence of incorporation into the recipient smooth muscle and differentiated with time (30). Enhanced bladder architecture and function was observed in small animal models upon ADSC injection (62) or in combination with an acellular scaffold (63). Moreover, in another study on a rat model, bladder acellular matrix (BAM) seeded with ADSCs showed enhanced detrusor muscle and neuronal regeneration, as well as improved bladder capacity (40). Furthermore, human ADSCs were differentiated into SMCs with smooth muscle inductive media and grown on PLGA scaffolds in a athymic rat model for bladder regeneration. The organ bath results demonstrated smooth muscle contraction of the seeded implants but not the acellular implants after 12 weeks *in vivo* (32). Moreover, human mature adipocyte derived cells could be differentiated into SMCs and contribute to the regeneration of the bladder wall (33).

Bone marrow derived MSCs (BM-MSCs) or stromal cells possess a self-renewal capacity and a potential to differentiate into the myogenic lineage. They are easily isolated due to their tendency to readily adhere to plastic culture dishes (64). Upon induction with TGF-beta1, they can differentiate to SMC, characterized by the expression of specific contractile proteins including alpha-SMA, calponin and SM-MHC (65). In some studies, BM-MSCs were evaluated as an alternative to bladder SMCs when healthy bladder tissue was unavailable (31). An *in vivo* study in a rat model showed that amniotic membranes seeded with BM-MSC could regenerate detrusor muscle and urothelium in the bladder wall but with no proper urinary bladder function (35). In a similar study performed by Chung et al., BM-MSCs seeded on SIS showed rapid cellular regeneration of bladder constituents morphologically, presenting a possible solution to overcome the fibrosis occurring in unseeded SIS bladder augmentations (29). A similar study in a canine hemicystectomy model using BM-MSC seeded SIS for augmentation demonstrated effective bladder regeneration with solid smooth muscle bundles throughout the graft (31). Although a few studies showed formation of smooth muscle using BM-MSCs in preclinical studies, its clinical application in bladder engineering is limited, due to the low isolation yield, difficulties in harvesting and expansion, and last but not least the painful collection procedure.

## Biomaterials

The complex anatomy and function of the urinary bladder pose unique challenges for the selection of scaffolds, cell types and cell sources for its bioengineering. The scaffold plays a key role in tissue regeneration and in re-establishing the biological function of the bladder tissue. Among the characteristics of the biomaterial determining the success of tissue regeneration are biocompatibility, biodegradability and scaffold architecture (66). In addition, an ideal scaffold for bladder TE should provide a microenvironment that promotes cell adhesion and a tissue organization similar to the native tissue (3, 67–71). Furthermore, the construct should serve as a barrier to urine, to protect the underlying tissue from the cytotoxic urine (72–74). It should display appropriate mechanical properties to sustain the mechanical forces necessary for bladder filling and emptying. Furthermore, the scaffold must be biodegradable at the proper rate to optimize integration into the bladder without triggering inflammation and foreign body reaction (70, 75). A special challenge ensuring regeneration and long term survival of the tissue *in vivo* is an adequate vascularization allowing for adequate oxygenation and nutrition of the regenerating tissue (76, 77). Scaffold materials for urologic tissue regeneration that are currently being investigated and have shown promise in clinical applications are mainly naturally or artificially derived biodegradable materials.

Acellular matrices are chemically and mechanically decellularized matrices such as BAM (78) and porcine SIS (79, 80). These collagen-based scaffolds have the advantage to maintain inherent bioactivity and feature the tridimensional architecture of the native tissue (81). The acellular matrices have been applied both preclinically (22, 79, 82, 83) and clinically (8, 84) with different outcomes (8, 23, 85). Major disadvantages of natural acellular matrices are the variability in physical and biochemical properties among batches (86, 87), the alterations of the physiological environment due to decellularization and sterilization processes (71, 88) and the possible triggering of an immune response (83, 89).

Similar to acellular matrices, naturally-derived polymers like collagen or silk, produced in a number of configurations and densities, provide distinct properties mimicking the structural and mechanical properties of native tissue extracellular matrix (ECM) while being biodegradable. They have shown great promise in a number of models of TE for regenerative medicine in numerous medical applications (90–96).

Collagen Type I, a ubiquitous structural protein, has been studied extensively as a possible scaffold in soft tissue TE applications (97). Collagen has already been approved by the FDA (Food and Drug Administration). It is bioactive, does not provoke immune responses and can easily be extracted from animal and human tissues (90, 92). However, hydrogel scaffolds from collagen offer only limited mechanical strength and different methods such as crosslinking (98), ultracentrifugation (99), or evaporation methods (100) are needed to produce high concentration collagen scaffolds. More recently plastic compression of the collagen hydrogel has been applied in different fields of TE and regenerative medicine e.g., for urinary bladder regeneration (92, 93). In this cell-independent technique developed by Brown et al. (101) and (102), excess water of the collagen hydrogel is removed via mechanical compression. This enables the fabrication of denser and stronger 3D nano- and micro-scale structures as compared to conventional gels (101). Promising preclinical results with compressed collagen scaffolds in TE suggest a potential for these constructs to be used as scaffolds for bladder tissue regeneration (92).

Silk fibroin (SF), another naturally derived material, has been proposed as biomaterial for soft tissue engineering owing to its versatility and biocompatibility (91, 103). Derived from Bombyx mori cocoons, this protein based polymer addresses many of the mechanical characteristics required for urologic TE applications (91, 96). Preclinical research involving SF scaffolds in urinary tissue regeneration has been encouraging as robust regeneration of smooth muscle and urothelium have been demonstrated (94, 95). SF scaffolds in combination with seeded bladder or mucosal cell populations are a promising strategy for engineering of functional urethral tissues (96).

Synthetic polymers are rapidly gaining ground as scaffold materials. In urologic TE, biodegradable synthetic biomaterials with appropriate mechanical properties for soft tissue regeneration such as poly (lactic-co-glycolic acid) (PLGA), polyurethane (104, 105), and poly(ε-caprolactone)/poly (L-lactic acid) (PCL/PLLA) (106) have found their application. Their main advantage is the manufacturing process which allows for suitable features of micro-nanostructure, strength and degradation in a constant quality and even on a large scale. Problems related to tissue harvesting are avoided with the use of these materials. However, none of these materials convinced *in vivo*. Disadvantages of synthetic polymers are their biological inertness and the lack of the molecular signals that are relevant for directing cell activity and fate. Furthermore, they can induce foreign body reaction, and degradation may produce acidic byproducts that may affect the local microenvironment of the regenerating tissue, causing inflammation and cell death (107, 108). Unfortunately, no single biomaterial or cell source provides all the desirable properties for successful urological tissue regeneration. Current technologies in bladder TE have been hampered by an inability to efficiently initiate blood supply to the graft, ultimately leading to complications that include graft contraction, ischemia, and perforation. These deficiencies therefore necessitate the evaluation of new strategies combining the gained knowledge to closer fulfill these requirements.

## Strategies to Succeed

The reason for tissue engineering a whole or partial bladder is to be able to deliver a functional substitute. Since the bladder wall is subjected to mechanical forces during filling and emptying cycles (109) selection criteria for cells and biomaterials are specific. Furthermore, in the pediatric population, a living functional tissue replacement with a good growth potential and a long life span is of main concern. To this end, the use of adult stem cells is given the most attention. Ideal stem cells need to be accessible with minimal invasiveness, have the ability to expand in a short period, and maintain a stable phenotype, while not changing during *in vitro* culturing but having a high differentiation potential. Therefore, the type, quality and quantity of stem cells for bladder engineering are critical factors.

Besides the already mentioned ESC (110), ADSC (58), and BM-MSCs (29) an other suitable stem cell candidate for urological tissue reconstruction are USCs. They can be isolated from voided urine within 24 h after urine collection (34, 111). USCs show MSCs characteristics and can be differentiated to SMCs, expressing all SMC lineage specific markers (111) with contractile function comparable to native SMCs (112). Originating in the urinary tract system, USCs are suggested as a good stem cell source for bladder TE with the benefits of simple, safe, low-cost and non-invasive collection technique (34). It was demonstrated that a USCs can differentiate in porous bacterial cellulose scaffolds, which may assist in the development of an engineered urinary conduit (34). Furthermore, it was shown that human USCs seeded scaffold-heparin-bFGF grafts improved biocompatibility, increased bladder capacity and compliance, as indicated by smooth muscle and urothelium layer in a partial cystectomy rat model (39).

In addition, a few studies showed that endometrial stem cells, which are of mesenchymal origin, can differentiate to SMCs and are suitable for bladder engineering (113). These cells can be harvested from the endometrium by two methods: either by an endometrial biopsy from the uterus or by collection of menstrual blood. In contrast to bone marrow and adipose tissue cells, for which at least a local anesthesia is required, these cells can be harvested without any anesthetic procedure (114). Furthermore, endometrial stem cells could also differentiate into urothelium using keratinocyte and epithelial growth factors, and in combination with 3D-silk-collagen they could serve as a suitable scaffold for building urinary bladder wall in females (115). However, endometrial-derived stem cells have not yet been used in any *in vivo* study.

Recent reports have shown that induced pluripotent stem cells (iPSCs) may answer the need for alternative cell sources for bladder regeneration. The iPSCs are reprogrammed, terminally differentiated somatic cells which have developed ESC-like cells characteristics following expression of various pluripotency transcription factors (116). Theoretically, iPSCs can produce an unlimited number of differentiated cells for autologous cell therapies (117). With this approach a patient's cells may be directed to become iPSCs and subsequently to differentiate and repair tissue. However, safety and efficiency is still under investigation. SMCs generated from iPSCs were shown to acquire contractile features and express contractile proteins (118, 119). Moad et al., for the first time, generated iPSCs derived from human urinary tract cells (bladder and ureter) which offers a potential for bladder engineering and *in vitro* studies (49). However, there are continuing concerns regarding induction of tumors by iPSCs. Currently discussed solutions include modifications in induction methods toward virus-free, transgene-free reprogramming and xeno-free systems (120, 121). In addition, the use of iPSCs requires an appropriate protocol for efficient *in vitro* differentiation, and in order to address safety issues its effect should be tested *in vivo*.

Current studies have shown that autologous cells in combination with biomaterials are the best options for bladder engineering. In addition, the construction of a three-dimensional scaffold *in vitro* before *in vivo* implantation would facilitate the terminal differentiation of the cells *in vivo*. The optimization techniques such as co-culture of different cell types and predifferentiation before implantation showed improved cells survival *in vivo* (122). Son et al. demonstrated that human dental pulp stem cells co-cultured with bladder derived SMCs or in a SMCs-conditioned medium with the addition of the transforming growth factor beta 1 (TGF-β1) can differentiate efficiently into bladder specific SMCs. This approach can be used as a less invasive alternative to harvest stem cells for smooth muscle regeneration and for bladder engineering (123). De-differentiation of SMCs from a contractile phenotype to a synthetic phenotype, which is characterized by SMCs hypertrophy and fibrosis is a known problem in bladder engineering. Methods to maintain the cell phenotype include cell culture microenvironment, the use of growth factors, the optimization of biomechanical and surface properties of the biomaterials and mechanical stimulation (47, 124).

The fabrication of hybrid or composite scaffolds consisting of at least two different biomaterials ideally allows the combination of the positive characteristics of the different compounds and even to develop new biomaterials with a wider range of physicochemical properties (36, 71). The use of hybrids of BAM and synthetic polymers has been described by our own group among others. We developed a bilayered scaffold by direct electrospinning of PLGA (36, 38) or Polyurethan microfibers (41) onto the luminal side of a BAM and demonstrated, that these scaffolds seeded with bladder SMCs supported the regeneration of a multi-layered bladder wall consisting of urothelium, lamina propria, and detrusor muscle resembling native control bladder in rats. Ajalloueian et al. combined CC hydrogels with electrospun PLGA sheets and studied the effect of different fibrillary densities on fibroblast performance (125). They showed that by decreasing the collagen content of CC hydrogel, not only a better cell environment and optimal mechanical properties are achieved, but also the application costs of this biopolymer are reduced. Another method to combine the physical properties of synthetic polymers with the biochemical as well as molecular characteristics of naturally derived scaffolds is the blending of natural and synthetic polymers as for example described by Moshfeghian et al. (126). They evaluated the formation of chitosan–PLGA blend matrices using controlled-rate freezing and lyophilization technique. By altering the freezing conditions they were able to control pore morphology and degradation kinetics of the scaffold with a positive influence on SMC spreading and colonization *in vitro*. Franck et al. produced a silk-based biomaterial coated with ECM (collagens or fibronectin), blending more than one naturally derived polymer to synthesize scaffolds for bladder tissue engineering (119). This composite scaffold was shown to be biocompatible and to support primary cultures of bladder UC, SMC, and pluripotent stem cell adhesion, proliferation, and differentiation. Such approaches can be adapted to a number of characteristics that are appropriate for bladder augmentation including mechanical properties, permeability, pore size, degradation characteristics, and biological activity (71).

The concept of natural self-assembly of cells differs from all other TE techniques that use pre-formed synthetic scaffolds. This innovative scaffold-free technique relies on the ability of cells to produce and assemble their own ECM (127). Initially introduced for skin TE (128), this approach also enabled the reconstruction of other tissues such as blood vessel, heart valve, cornea, adipose tissue, vaginal mucosa, and urinary tissues (129). In a recent study, Orabi et al. were able to produce a multi-layered construct with histological and molecular properties similar to native tissue *in vitro*. For this approach, they used bladder-specific stromal cells from the lamina propria co-cultured with UCs or SMCs (130). However, the self-assembly technique still needs to be investigated for urologic tissue regeneration *in vivo*.

To improve the outcome of bladder regeneration, scaffolds can be functionalized with growth factors, creating a microenvironment that simulates the integration of the tissue engineered constructs (131, 132). Physiologically, growth factors are components of the ECM, which are actively released after injury. They play a crucial role in tissue repair and the prevention of fibrosis. The therapeutic use of recombinant growth factors is based on the hypothesis that through appropriate signaling they induce and/or accelerate the healing process. Several growth factors have been identified as important in the development of functional urological tissue (133), mainly vascular endothelial growth factor (VEGF) and nerve growth factor (NGF) (134, 135). The use of VEGF alone or in combination with NGF resulted in improved bladder wall regeneration and angiogenesis (134–136). When using stem cells, the presence of appropriate growth factors is essential for cell differentiation (137, 138). In most cases, incorporation of biologically active molecules into the scaffold material has been aimed at rapid restoration of vascular networks to maintain tissue viability and long-term survival. Growth factors are considered to be critically important modulators during all phases of tissue regeneration (71). To provide an effective delivery of growth factors, some challenges must be overcome. Because of the high instability of growth factors *in vivo*, various slow-release devices of natural, synthetic and composite materials have been designed (139, 140). Biomaterials with degradable porous reservoir structures or pre-encapsulated microspheres have been used to control effective targeting (141). For an effective long-term delivery, growth factors can be encapsulated in biodegradable polymers, such as poly(lactic-co-glycolic acid) (PLGA) or poly-L-lysine (PLL) (142, 143). Those systems are designed to release the loaded protein in a sustained manner following the degradation of the polymer. To improve the controlled delivery, Layman et al. developed ionic-albumin microspheres that allow the time-controlled release of two growth factors (144).

Nanoparticles have not only been used for controlled delivery of bioactive molecules and growth factors, in TE they have been used in order to improve the mechanical and biological performance of the regenerated tissue (145). For example nanoparticles can play a vital role in enhancing the mechanical properties of the scaffold as shown in skin TE, where the tensile strength of collagen or silk fibroin was improved by using TiO2 (146) or hydroxyapatite nanoparticles, respectively (147). Furthermore, nanoparticles can mimic the natural nanostructure of ECM components of tissues, and therewith influence cellular activities such as adhesion, growth and differentiation of stem cells (148, 149). Although the use of nanoparticles recently made an enormous progress, *in vivo* experimentation to verify the successful results from *in vitro* studies (150) are still needed.

The establishment of a functional vascularization represents one of the major challenges for the implementation of TE applications in clinical practice. The survival of larger and complex tissue substitutes after implantation depends on the rapid development of an adequate vascularization. Furthermore, vascularization is a major prerequisite for a complete restoration of organ structure and functionality. Classical approaches to promote vascularity in tissue substitutes focus on the stimulation of vascular ingrowth into tissue constructs by optimizing the material properties of scaffolds (38, 151, 152) or by enriching implants with proangiogenetic factors (153–155). A promising approach is the incorporation of growth factors which can be released in a time-dependent manner at the implantation site. Therefore, various slow-release devices of natural, synthetic, and composite materials have been designed (156, 157). An additional approach to supply growth factors is the use of transfected cells, which overexpress angiogenic factors (158).

Prevascularization of tissue constructs with networks of capillaries aims to accelerate functional anastomosis with host tissue upon implantation. *In vitro* prevascularization of thicker constructs and the connection to the host vasculature *in vivo* is essential to guarantee immediate supply to the cells within the construct. These requirements determine the success of the applied transplant (158, 159). However, angiogenesis in a large avascular graft *in vivo* does not occur fast enough to avoid hypoxic conditions (160). This innovative approach basically aims at the generation of preformed microvascular networks in tissue constructs prior to their implantation by co-culturing endothelial cells (ECs) with supporting cells (161). The co-culture approach is the most biomimetic option, which can be achieved by growing ECs with mural cells, such as fibroblasts (162) or SMCs (163) or MSCs (164). Also the self-assembly technique showed promising results with endothelialized substitutes for skin (165) and urethral reconstruction (37). After implantation, these networks can then be rapidly perfused with blood by inosculation with the surrounding host microvasculature (166) or by surgical anastomosis of feeding and draining blood vessels (167, 168). This enhances earlier vascularization of the graft, thus potentially decreases the risk of ischemia, necrosis and fibrosis and enhances graft regeneration and thereby long term function. As the feasibility of engineering blood vessels in bladder grafts becomes reality, inosculation and prompt nourishment of grafts upon transplantation will further potentiate the clinical use of bioengineered bladder tissue (73). However, mimicking natural vascular architecture and rebuilding microvascular networks *in vitro* is still challenging and limits clinical applications. These promising achievements lead to further advancement of these prevascularization concepts and their adaptation to individual therapeutic interventions will markedly contribute to a broad implementation of TE applications in clinical practice.

## Outlook and Clinical Translation

Urinary diversions made from engineered bladder tissue would remove the need for bowel tissue for bladder reconstruction. An off-the-shelf bladder tissue would revolutionize reconstructive urology and would allow a substantial reduction in morbidity and improve the long term outcome of bladder augmentation, especially in the pediatric patient. With recent advances in isolating, growing, and differentiating host stem cells, an increased understanding of the cell niche required to maintain the artificial tissue, and novel techniques for the generation of an intact blood supply, it appears that the major elements for the engineering of a functional bladder wall are achievable. Despite impressive progress in the field of bladder TE over the past decades, the successful transfer of these approaches into clinical routine still represents a major challenge. Large animal trials are necessary to confirm the applicability of the approaches in a model similar to the growing human organism to meet the special needs of this patient group.

As discussed in this review there are several strategies to overcome the hurdles of TE which lead to new approaches in bladder regeneration. In order to improve the bladder engineering for clinical application we suggest further unifications of the strategies and approaches including a collaborative effort of experts of different fields. With its complex nature and distinct mechanical properties, the development of a next generation bioengineered bladder tissue requires the combined knowledge and techniques of material science and cell biology to be successful in future clinical application.

## Data Availability

The datasets for this manuscript are not publicly available because its a review. Requests to access the datasets should be directed to maya.horst@ksipi.uzh.ch.

## Author Contributions

MH and SS wrote the manuscript. DE and RG authors contributed to the final version of the manuscript.

### Conflict of Interest Statement

The authors declare that the research was conducted in the absence of any commercial or financial relationships that could be construed as a potential conflict of interest.
